# Automation in urinalysis: sample and data management, and quality control

**DOI:** 10.1155/S1463924686000275

**Published:** 1986

**Authors:** Giuliano Barbaresi, Maria Luisa Gozzo, Cecilia Zuppi

**Affiliations:** lstituto di Fisiologia Umana Laboratorio di Chimica Clinica Università Cattolica del Sacro Cuore Roma Italy


Journal of Automatic Chemistry/*Journal of Clinical Laboratory Automation*, Vol. 8, No. 3 (July–September 1986), pp. 142–146

### Automation in urinalysis: sample and data management, and quality control

**Giuliano Barbaresi, Maria Luisa Gozzo and Cecilia Zuppi**

*Istituto di Fisiologia Umana, Laboratorio di Chimica Clinica, Università Cattolica del Sacro Cuore, Roma, Italy*

### Introduction

Qualitative urinalysis, accomplished by means of commercial reagent strips, is simple, inexpensive and rapid, but when manually performed it is based on subjective discrimination between subtleties of hue and gradations in opacity. Inter-observer differences in test strip interpretation have been shown to be important causes of variability of results.

The introduction of automatic analysers, based on reflectance photometry, overcame the difficulty of subjective interpretation, but posed problems of sample and data management and required more sophisticated quality control systems.

This paper describes procedures for sample and data management and for a specific quality control programme.

### Material and methods

*Urine analysers:* Automated Urine Analyser Hi-Speed Aution Analyser Model HS-7 and Digital Urinometer Biovation linked on line with the DP-8N Buffer memory (all by Kyoto, Daiichi Kagaku Co. Ltd, Japan, supplied in Italy by Menarini of Firenze).

*Hardware:* Display CRT Terminal DSM 6680, Minicomputer P6060 with a 32 Kbyte ROM and 400 Kbyte RAM in floppy disks equipped with standard interface, Printer PR 1230 (130 characters/s), and OPR 1830 Wand Automatic Optical Reader for OCR-B characters (all by Olivetti ing.C., Spa, Ivrea, Italy).

*Reagents:* Urine reagent strips Uriflet 7A DIC, Check Sample Set (used for calibration control of Aution analyser) and Rifra-Chek Solutions for calibration and control of Urinometer lot 040 (all by Kyoto, Daiichi, Kagaku Co. Ltd, distributed in Italy by Menarini of Firenze). Urine Controls: Kova Trol 1, Kova Trol 2, and Kova Trol 3: Human Urine (dried) manufactured by ICL Scientific, 18349 Euclid, Fountain Valley, California 92708, USA (distributed in Italy by Boehringer Biochemia Robin Diagnostic Division, Milano).

In this study, fresh urine samples from hospital and out-patient clinics were used.

*Software*

The programs in Basic were written by laboratory staff. A complete listing of the programs is available from the authors (see figure 1).

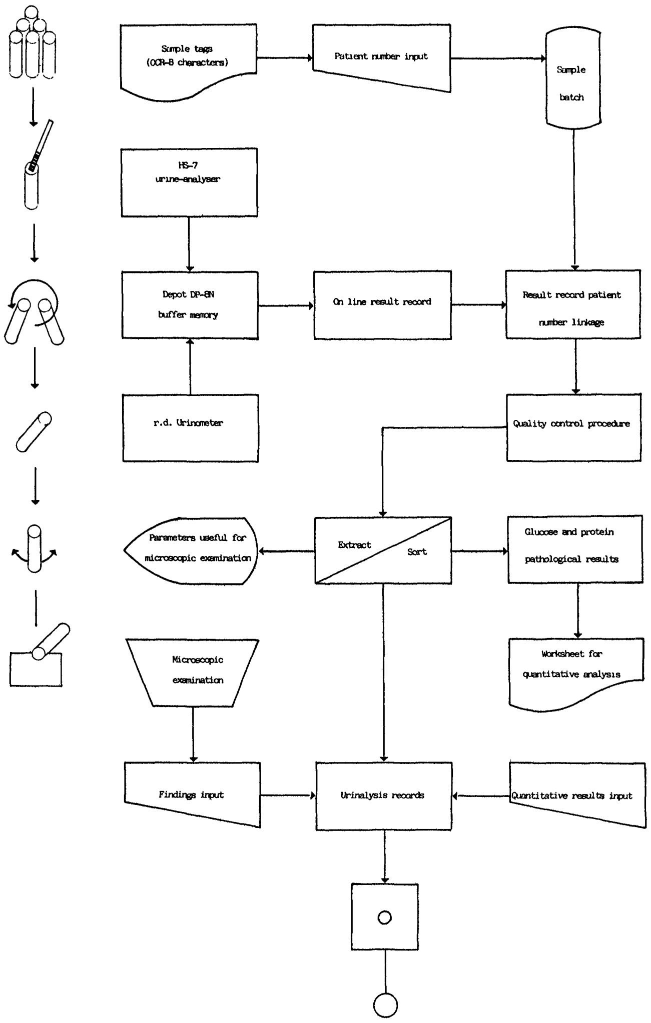

*Figure 1. Flow chart of urine samples and data management.*

Urine samples, in cuvettes distinguished by yellow caps, are randomly analysed as they are delivered to the laboratory. Using the same random sequence, patient identification numbers, printed on containers tags in OCR-B characters, are entered in the minicomputer automatically (optical reader) or manually (keyboard). The seven-reagent strip (pH, glucose, protein haemoglobin, ketone bodies, bilirubin and urobilinogen) dipped in the urine specimen, is placed on the turntable of the HS-7 analyser. The strip is carried automatically to the optical

*Table 1. Scale for reporting results of urinary sediment examination.**

| Sediment concentration 1 : 10 | Neg. | Occasional | 1+ | 2+ | 3+ | 4+ |
|---|---|---|---|---|---|---|
| Erythrocytes (high power field) ×440 | 0 | Less than 4 | 4–8 | 8–30 | 30 | Packed field |
| White cells (high power field) ×440 | 0 | Less than 5 | 5–20 | 20–50 | 50 | Packed field |
| Casts and abnormal crystals (low power field) ×100 | 0 | Less than 1 | 1–5 | 5–10 | 10–30 | 30 |

* From M. Bradley *et al.*: Examination of urine, in Henry, J. B. *Clinical Diagnosis and Management by Laboratory Methods*, 16th edn (W. B. Saunders Company, Philadelphia, London and Toronto, 1979), p. 613.

section for reflectance measurement and the results are stored in DP-8N buffer memory.

Urine samples are then spun at 2000 r.p.m. for 5 min using a standard bench centrifuge. The clear supernatant (about 90% of the total specimen) is decanted in the Urinometer for relative density (r.d.) refractometric determination. Relative density results are also stored in the DP-8N. When the analysis is complete the results generated by the HS-7 and Urinometer are transferred to the minicomputer and linked to relevant patient numbers.

A drop of the pellet, resuspended in the remaining supernatant, is placed on a slide for microscopic examination. As an aid to correct interpretation of each microscopic test, the respective data of pH, haemoglobin and protein are displayed in sequence on the CRT terminal. The findings are then fed into the minicomputer using a three-digit codex: the first two digits identifying the elements, and the last one quantifying them (see table 1).

At the same time, glucose and protein abnormal results are sorted and worksheet for quantitative determinations are printed. Quantitative results are then entered by keyboard. Records of complete urinalyses are transferred to the hospital's data processing centre (HDPC) by means of a floppy disk for personalized report print-out.

### Quality-control system

Quality control (see figure 2) includes inspection of the results of urine controls at three concentration levels analysed in the sample batch after 30 specimens, and retrospective analysis of patient result distribution once the analyses are complete.

Two procedures are provided for physicochemical (pH, s.g.) and chemical parameters (glucose, albumin, haemoglobin, ketones, urobilinogen, bilirubin). Results of physicochemical parameter determinations are subdivided into 10 frequency classes. Next the percentage frequency and the percentage cumulative frequency of each class are calculated and logit transformed. Finally, the patient distribution mean and 95% limits of the normal range are obtained by a plot of logit transformed

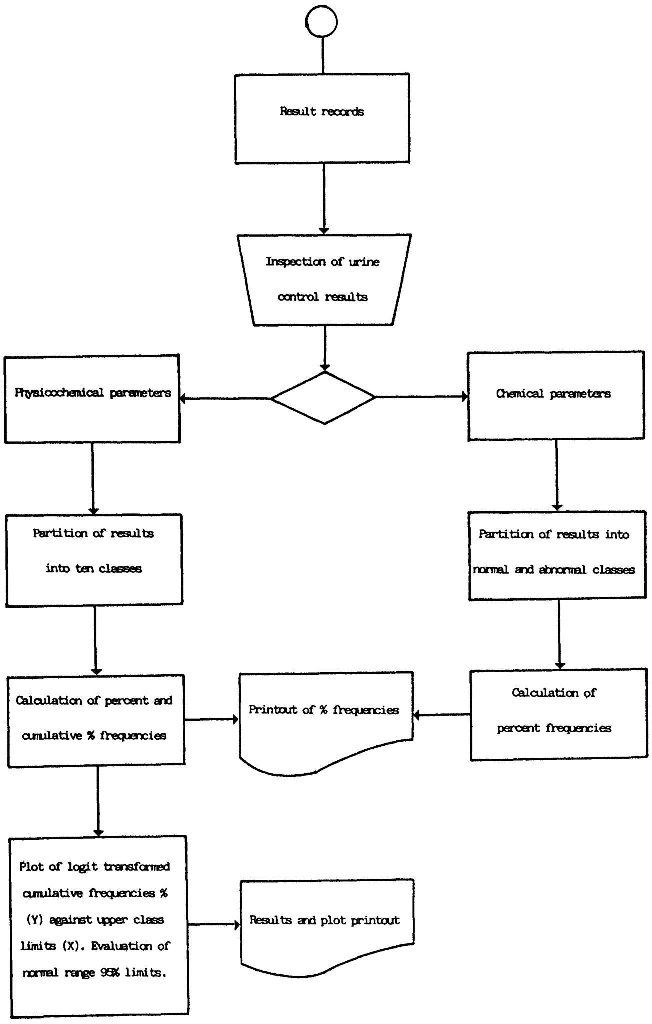

*Figure 2. Flow chart of quality-control procedure.*

data ($y$) against upper class limits ($x$) (see figure 3). The procedure for chemical parameters calculates the frequency in per cent of normal and abnormal results.

Both procedures are applied to the hospital patients, to the out-patients and to the cumulative results. Finally the program prints-out all calculated parameters (see figure 3).

■[18 10 84] URINALYSIS STATISTICS [All Patients (n.= 194 )]

| | | | | | | | | | | |
|---|---|---|---|---|---|---|---|---|---|---|
| pH | 4.5 | 5.0 | 5.5 | 6.0 | 6.5 | 7.0 | 7.5 | 8.0 | 8.5 | 9.0 |
| Frequency | 0 | 8 | 78 | 58 | 27 | 11 | 9 | 3 | 0 | 0 |
| Frequency% | 0.0 | 4.1 | 40.5 | 29.8 | 13.9 | 5.6 | 4.6 | 1.5 | 0.0 | 0.0 |
| GLUCOSE | Norm | Path | PROTEIN | | Norm | Path | HEMOGL. | | Norm | Path |
| Frequency | 191 | 3 | Frequency | | 178 | 16 | Frequency | | 171 | 23 |
| Frequency% | 98.5 | 1.5 | Frequency% | | 91.8 | 8.2 | Frequency% | | 88.2 | 11.8 |
| BILIRUBIN | Norm | Path | KETONES | | Norm | Path | UROBIL. | | Norm | Path |
| Frequency | 190 | 4 | Frequency | | 188 | 6 | Frequency | | 184 | 10 |
| Frequency% | 98.0 | 2.0 | Frequency% | | 97.0 | 3.0 | Frequency% | | 94.9 | 5.1 |
| r.d. | 1005 | 1010 | 1015 | 1020 | 1025 | 1030 | 1035 | 1040 | 1045 | 1050 |
| Frequency | 10 | 22 | 54 | 44 | 38 | 20 | 4 | 1 | 0 | 0 |
| Frequency% | 5.1 | 11.3 | 28.5 | 22.7 | 19.6 | 10.3 | 2.0 | 0.5 | 0.0 | 0.0 |

■[18 10 84] URINALYSIS STATISTICS [In Patients (n.= 111 )]

| | | | | | | | | | | |
|---|---|---|---|---|---|---|---|---|---|---|
| pH | 4.5 | 5.0 | 5.5 | 6.0 | 6.5 | 7.0 | 7.5 | 8.0 | 8.5 | 9.0 |
| Frequency | 0 | 3 | 43 | 31 | 16 | 8 | 7 | 3 | 0 | 0 |
| Frequency% | 0.0 | 2.7 | 38.8 | 27.9 | 14.4 | 7.2 | 6.3 | 2.7 | 0.0 | 0.0 |
| GLUCOSE | Norm | Path | PROTEIN | | Norm | Path | HEMOGL. | | Norm | Path |
| Frequency | 109 | 2 | Frequency | | 99 | 12 | Frequency | | 95 | 16 |
| Frequency% | 98.2 | 1.8 | Frequency% | | 89.2 | 10.8 | Frequency% | | 85.6 | 14.4 |
| BILIRUBIN | Norm | Path | KETONES | | Norm | Path | UROBIL. | | Norm | Path |
| Frequency | 107 | 4 | Frequency | | 105 | 6 | Frequency | | 102 | 9 |
| Frequency% | 96.4 | 3.6 | Frequency% | | 94.6 | 5.4 | Frequency% | | 91.9 | 8.1 |
| r.d. | 1005 | 1010 | 1015 | 1020 | 1025 | 1030 | 1035 | 1040 | 1045 | 1050 |
| Frequency | 7 | 18 | 35 | 22 | 22 | 7 | 0 | 0 | 0 | 0 |
| Frequency% | 6.3 | 16.2 | 31.6 | 19.8 | 19.8 | 6.3 | 0.0 | 0.0 | 0.0 | 0.0 |

■[18 10 84] URINALYSIS STATISTICS [Out Patients (n.= 83 )]

| | | | | | | | | | | |
|---|---|---|---|---|---|---|---|---|---|---|
| pH | 4.5 | 5.0 | 5.5 | 6.0 | 6.5 | 7.0 | 7.5 | 8.0 | 8.5 | 9.0 |
| Frequency | 0 | 5 | 35 | 27 | 11 | 3 | 2 | 0 | 0 | 0 |
| Frequency% | 0.0 | 6.0 | 42.3 | 32.5 | 13.2 | 3.6 | 2.4 | 0.0 | 0.0 | 0.0 |
| GLUCOSE | Norm | Path | PROTEIN | | Norm | Path | HEMOGL. | | Norm | Path |
| Frequency | 82 | 1 | Frequency | | 79 | 4 | Frequency | | 76 | 7 |
| Frequency% | 98.8 | 1.2 | Frequency% | | 95.2 | 4.8 | Frequency% | | 91.6 | 8.4 |
| BILIRUBIN | Norm | Path | KETONES | | Norm | Path | UROBIL. | | Norm | Path |
| Frequency | 83 | 0 | Frequency | | 83 | 0 | Frequency | | 82 | 1 |
| Frequency% | 100.0 | 0.0 | Frequency% | | 100.0 | 0.0 | Frequency% | | 98.8 | 1.2 |
| r.d. | 1005 | 1010 | 1015 | 1020 | 1025 | 1030 | 1035 | 1040 | 1045 | 1050 |
| Frequency | 3 | 4 | 19 | 22 | 16 | 13 | 4 | 1 | 0 | 0 |
| Frequency% | 3.6 | 4.8 | 23.1 | 27.2 | 19.5 | 15.8 | 4.8 | 1.2 | 0.0 | 0.0 |

*Figure 3 continued over*

```

■(18 10 84) PHYSICOCHEMICAL PARAMETERS : MEANS AND NORMAL RANGES

               ==================================================

pH    : 2.5%= 4.8      50%= 5.8      97.5%= 6.9      (All Patients)

r.d.  : 2.5%= 1004     50%= 1016     97.5%= 1027

pH    : 2.5%= 4.9      50%= 5.9      97.5%= 7.0      (In Patients)

r.d.  : 2.5%= 1004     50%= 1013     97.5%= 1022

pH    : 2.5%= 4.8      50%= 5.7      97.5%= 6.6      (Out Patients)

r.d.  : 2.5%= 1006     50%= 1017     97.5%= 1029

```

*Figure 3. Daily report of urine patient result statistics.* ■ Date.

### Results and discussion

Urine analysis yields a great deal of clinical information for diagnosis and management of renal or urinary tract diseases, and for the detection of metabolic and systemic diseases not directly related to the kidney. Complete urinalyses should include both biochemical and microscopical findings.

The technique for qualitative urinalysis using reagent strips is simple, inexpensive and is performed in most clinical laboratories. Automatic analysers have recently become available for reading reagent strips and for refractometric determination of r.d. These commercial analysers overcome the difficulties arising from subjective interpretations of results and from handling large volumes of samples. They also provide a complete personalized report by input of identification data (name, surname) and nosographic numbers. Thus using these commercial urinalysis systems in laboratories such as the authors', supported by a data-bank of HDPC which automatically daily supplies patient data, generates additional clerical work.

The authors propose a procedure that ensures rational employment of the analysers and supplies a complete report without overlapping with the HDPC. Urinalysis records are transferred, by floppy disks, to HDPC for a personalized and complete report print-out and storage in the central data bank.

Interesting features of the authors' system are the possibility of displaying physicochemical parameters as a support at the time of microscopical examination and the possibility of sorting glucose and protein pathological results for quantitative determinations. Little work on intra-laboratory urinalysis quality control systems has been reported if compared with quality-control programs available for other parameters in clinical chemistry, and the literature on inter-laboratory surveys shows, generally, a poor standard performance [1 and 2]. The reason for this is the difficulty of quantifying the accuracy and the precision of qualitative results. Reliable and stable urine controls are commercially available and routinely employed, but the criteria of interpretation of their results are unsatisfactory. It is generally accepted that any reaction grade one step higher or lower than the expected value of urine controls is sufficient [1]. The authors' experience indicates that this criterion alone might not ensure effective monitoring of urinalysis performance. In order to ensure that the urinalysis quality-control system was reliable and sensitive, statistical analysis of patient results was introduced, adapting control procedures routinely used in clinical chemistry [3, 4 and 5].

The distribution of patient results should be constant under stable analytical conditions and without population changes. At the authors' hospital the composition of the out-patient population is quite constant, and so it is possible to discriminate between the effects of population changes and those of analytical errors by separately considering hospitalized and out-patients. As far as the total number of patients is concerned, it can vary from a maximum of 300/day to a minimum of 150/day, the number of out-patients being constant around 70/day. The latter population is large enough not to influence the distribution [6].

The rationale for setting the interpretation criteria of daily patient distribution patterns is as follows.

For urinalysis qualitative chemical parameters it is assumed that, in a normal population, the percentage frequency of negative results should very close to 100%. So, a random error higher than 5% is unacceptable, and the percentage frequency of daily negative results cannot be lower than 95%. table 2 shows the mean percentage frequency of daily negative results for qualitative chemical parameters obtained under stable analytical

*Table 2. Mean percentage frequency of daily negative results in stable analytical conditions.*

| | All patients | In-patients | Out-patients |
|---|---|---|---|
| Glucose | 96% ± 1·9 | 96% ± 2·2 | 97% ± 2·0 |
| Protein | 86% ± 3·5 | 83% ± 4·3 | 94% ± 3·0 |
| Haemoglobin | 77% ± 4·3 | 71% ± 4·7 | 94% ± 3·2 |
| Bilirubin | 98% ± 2·0 | 98% ± 1·6 | 99% ± 1·1 |
| Ketones | 95% ± 2·4 | 93% ± 3·0 | 99% ± 1·4 |
| Urobilinogen | 95% ± 1·7 | 93% ± 2·2 | 99% ± 2·0 |

The reported values are the means ± SD calculated from 30 daily percentage frequencies under stable analytical conditions.

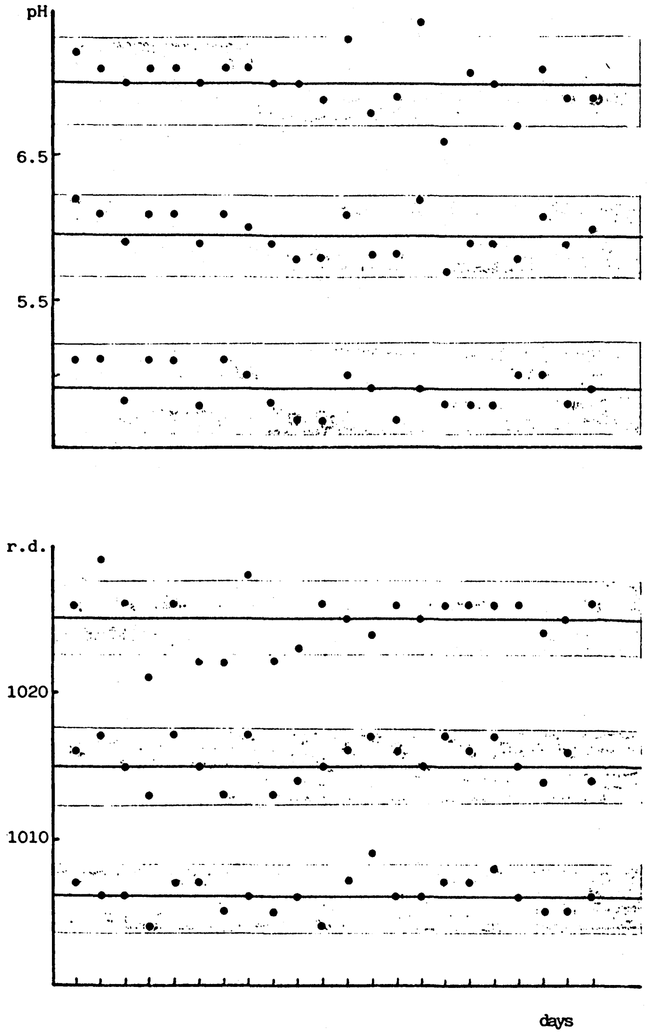

*Figure 4. Control charts for pH and r.d. of daily out-patients' results. The horizontal lines represent the true values for both daily means and 95% control limits. The acceptability ranges are marked by shading.*

conditions. The values for the out-patients are higher than for the hospital patients, and greater than 95% except for protein and haemoglobin. These findings may be explained by physiological trace amounts of protein and haemoglobin. Therefore, for these parameters, the mean frequency of daily negative results should not be lower than 90%. The percentage frequency of glucose negative results for out-patients is similar to that of hospital patients; a reason for this might be that hospital patients have a controlled diet. The constancy of analytical performance is then certified by regular behaviour of the daily negative results frequencies into 90% and 95% limits. For pH and r.d. it is believed that if the laboratory testing procedure is unstable, then the limits of normal range of out-patient population will shift. The evaluation of daily performance (figure 4) is accomplished by comparing daily means and normal limits with the mean value obtained during a period of 30 days under optimal analytical conditions. These values are then considered as the 'true values' in the daily control chart. The acceptability range (the shaded area in figure 4) is defined as $x \pm 1.96$ SD both for normal limits and for the mean. It would be impossible to adopt the standard error of the mean ($SE = SD/\sqrt{N}$) since this value is too small and lower than the instrument's resolution power. Figure 4 shows the typical behaviour of out-patients' daily results for pH and r.d.

The introduction of the system described in the authors' laboratory was able to solve the problems resulting from the urinalysis automation, concerning sample and data management and quality control. A more rational employment of analysers and a certified analytical performance was achieved.

### Acknowledgement

This work was supported by funds of CNR and MPI.

### References

1. Hoeltge, G. A. and Ersts, A., *Pathology*, **73** (1980), 403.

2. Shepard, M. D., Penberthy, L. A. and Fraser, C. G., *Pathology*, **14** (1982), 333.

3. Hoffmann, R. G. and Waid, E., *American Journal of Clinical Pathology*, **43** (1965), 134.

4. Reed, A. H., *Clinical Chemistry*, **16** (1970), 129.

5. Dixon, K. and Northon, B. E. *Clinica Chimica Acta*, **30** (1970), 453.

6 Whitehead, T. P. *Quality Control in Clinical Chemistry* (John Wiley Sons, Inc., 1977), 71.

